# Editorialets

**Published:** 1908-04

**Authors:** 


### Editorialets.

THE CAUSE AND PREVENTION OF RAPE, by the editor of this journal, published in our May, 1904, number, is reproduced herewith in response to numerous requests for it, the edition of the JOURNAL and reprints being long since exhausted. Interest in the subject has been revived by numerous able articles on the negro, recently published, notably the wonderful book of Dr. Shufeldt, a notice of which will be found in our editorial columns, and the address of Judge Norwood, of Savannah, Ga., in retiring from the bench. A copy of my paper will be mailed to any address on request with stamp enclosed. DR. DANIEL.

---

"YOUR journal (the 'Red Back') is always in demand and is inquired for at this office several days prior to its arrival. Abbott Alkaloidal Co., Chicago."

---

PAY YOUR STATE MEDICAL ASSOCIATION dues and dues to county society before it is too late, and keep up your membership. In default you will be dropped, and it is rather difficult and tedious to be reinstated.

---

THE TEXAS STATE BOARD OF MEDICAL EXAMINERS will hold next regular meeting for examination of applicants for license to practice medicine in Texas, at Waco, June 30. Dr. G. B. Foscue, Secretary, Waco, Texas.

---

AFTER THE PULLMANS.—State Health Officer Brumby has instituted suit at San Angelo, Texas, against the Pullman Car Company for failure to comply with the law requiring disinfection of sleepers. The penalty is from $20 to $200. Other suits will follow.

---

THE ANNUAL MEETING of the Texas State Medical Association will be held at Corpus Christi, May 13, 14, 15 prox. A splendid program has been arranged, as well as a series of social delights. Accommodations are ample and a few days' sojourn by the sounding sea will rejuvenate ye jaded doctor and make him feel equal to anything. Let us have a rousing attendance,—a record-breaker. For details as to rates, routes, hours of departure, see the official organ. Don't fail to pay your dues at once, or you will be down and out.

---

THE BATTLE CREEK SANITARIUM SYSTEM.—Dr. Kellogg, the founder and superintendent of this world-famous institution, has sent me a copy of a beautiful illustrated little book bearing the above title, which gives a complete history of the founding, rise, progress and success of the Battle Creek Sanitarium, together with a description of the numerous devices for the treatment of all kinds of diseases (contagious and infectious excepted). A copy can be had for the asking, stamp enclosed, by addressing Battle Creek Sanitarium, or Dr. J. H. Kellogg, Battle Creek, Mich.

---

THREE MORE OLD LANDMARKS GONE.—Dr. D. B. St. John Roosa, the founder of the New York Post-Graduate, died March 8th at his home in New York, aged 72; and Dr. Dudley D. Saunders, of Memphis, died of pneumonia, February 16, aged 72. Dr. Saunders was a distinguished Confederate surgeon and yellow fever expert, greatly beloved by all who knew him. Dr. J. M. Reuss died March 20th at the home of his son, Dr. J. H. Reuss, of Dallas, aged 84 years. Dr. Reuss was one of the pioneers of Texas and was well known throughout the State. He was a native of Wurzburg, Bavaria, and came to Texas with Prince Braunfels, settling at Old Indianola in 1845. After the storm and flood at Indianola, Dr. Reuss went to Cuero, where he established a drug store and became prominent also as a physician and surgeon.

